# Diversity and Resistance to Change: Macro Conditions for Marginalization in Post-industrial Societies

**DOI:** 10.3389/fpsyg.2018.00812

**Published:** 2018-06-01

**Authors:** Charles Lassiter, Vinai Norasakkunkit, Benjamin Shuman, Tuukka Toivonen

**Affiliations:** ^1^Department of Philosophy, Gonzaga University, Spokane, WA, United States; ^2^Department of Psychology, Gonzaga University, Spokane, WA, United States; ^3^Applied Defense Solutions, Columbia, MD, United States; ^4^UCL Institute for Global Prosperity, University College London, London, United Kingdom

**Keywords:** agent-based model, marginalization, resistance to change, diversity, globalization, cultural change

## Abstract

We argue that two society-level properties—resistance to change and diversity within a culture—significantly affect agents' degrees of marginalization, which is here defined as access to cultural knowledge and institutional means for accomplishing cultural goals. We develop an agent-based model using findings from Norasakkunkit et al. (Norasakkunkit and Uchida, 2011, 2014; Norasakkunkit et al., 2012). We found that varying the degrees of resistance to change and diversity affected similarities between the mainstream subculture and other subcultures, changes in subcultures over time, and the relative population proportion of each subculture. In particular, we found that high diversity and low resistance to change created the greatest cultural changes within the marginalized subculture over time and allowed for maximal growth of rebellious subcultures. Also, low diversity and high resistance to change allowed for maximal growth of the marginalized subcultures and the greatest overlap between the marginalized and mainstream subcultures. These have important implications for understanding the emergence and maintenance of marginalization in post-industrial societies.

## 1. Introduction

No one doubts that society-wide conditions affect the behaviors and interactions of individuals. And no one doubts that the behaviors and interactions of individuals affect society-wide conditions. But manipulating macro-level conditions to explore micro-level consequences is substantially harder. While a city might be a living laboratory for social scientists, it's not the kind of laboratory where easy adjustments can be made. Fortunately, developments in agent-based modeling enable simulation of micro and macro condition interactions, albeit in simplified models^1^. Agent-based models (ABMs) allow their creators to build agents and their environments, and then to lay down the rules by which they interact. They have been used to explore issues as diverse as opinion dynamics (Deffuant et al., 2002; Hegselmann and Krauss, 2002), consumer behavior (Izquierdo and Izquierdo, 2007), segregation (Sadoka, 1971; Schelling, 1971), consequences of testimonial norms (Zollman, 2015), and movements of stream fish (Railsback et al., 2005). We take up a suggestion from Kashima (2014, cf. Kashima, 2016), who notes that ABMs “can be used to examine the macro-level implications of micro-level cultural processes…” While the models are simplified versions of reality, they enable manipulation of macro conditions in a way not possible in the real world.

The macro-level condition we focus on is marginalization in post-industrial society. “Marginalization” here refers to the minimal access to institutional means to accomplish cultural goals. Marginalized groups have restricted access to resources like education and healthcare for achieving their aims. In the model, we manipulate two macro-level conditions that affect marginalization: diversity and resistance to change (to be discussed in greater detail in the next section). Modifying these macros change micro-level interactions. Repeated micro-level interactions generate macro-level conditions. Macro conditions that are most conducive to the growth of marginalized populations are high institutional resistance to change and low cultural diversity (Zielenziger, 2006).

## 2. Background and hypotheses

### 2.1. Background: agents

According to the classic sociologist Merton's (1938) model of anomie, there are five possible groups in society, four of which represent greater-and-lesser marginalized groups.^2^ They are the conformists, the innovators, the ritualists, the retreatists, and the rebels.

Conformists are the odd ones out, the only non-marginalized group. They are individuals who have embraced mainstream cultural goals and have full access to the institutional means to accomplish them. For example, if the cultural goals are to find a secure job, buy a home, and raise a family, the conformists have access to good education, financial means, family support, and the social networks necessary to accomplish those goals. Thus, the conformists represent the mainstream of society and benefit from dominant institutional practices.

In contrast, the individuals who do not have access to these means but still embrace cultural goals may find alternative, unconventional, or even illegal means to accomplishing those goals. These are the innovators. For example, an American who commits a white-collar crime in order to maintain his upper class lifestyle is an innovator. He pursues cultural goals of affluence but not by culturally approved routes. Then there are those who have access to legitimate means, but not sufficiently enough to accomplish cultural goals. These are the ritualists. For example, part-time or temporary workers with no benefits go through the motions of everyday work. But they do not reap many of the benefits that conformist can take for granted (e.g., retirement savings). The fourth group consists of those who have little access to institutional means and do not embrace dominant cultural goals. They are retreatists; they retreat from participating in society. In the UK and Japan, for example, retreatists are often known as NEETs: “Not in Employment, Education, or Training.” Alternatively, retreatists can also be involved in highly unstable, low-skilled, and precarious work activities, such as passing out fliers on the street or gathering empty cans or bottles in exchange for a cash refund. Finally, some individuals will reject legitimate means and conventional cultural goals in place of their own goals which are accomplished by devising their own means. These are the rebels in Merton's scheme. Occupants of utopian communes in 19 and 20th America are rebels. They drop out of conventional society to pursue goals of their own choosing. Rebels also include those who leave their society to access goals and means not available there.

More recently, Toivonen et al. (2011) have reworked Merton's model of anomie by collapsing the marginalized groups who fall into Merton's ritualists and retreatists categories into one group called “deviants.” This was done to suggest that both ritualists and retreatists appear as a spectrum of individuals and behaviors that deviate from mainstream cultural norms and practices. Thus, the deviants are either marginalized or at risk of being marginalized in their own society. To be marginalized is to be a deviant.

Additionally, Toivonen et al. (2011) identified a class of individuals called “quiet mavericks.” They usually have the access and resources to be conformists but go above and beyond conforming to mainstream goals and values by being agents of change for their society. Quiet mavericks tend to be innovative about quietly rebelling against mainstream practices in their society. They create alliances with mainstream actors to establish new paths for future conformists. They are the social entrepreneurs and the globally-minded pioneers who creatively adopt new ideas and technologies to create solutions meeting new demands. Essentially, quiet mavericks integrate the skill sets of conformists and of rebels. They can also flexibly switch back and forth between those mindsets, as needed. The quiet mavericks were created to fill the void in Merton's model as it is applied to the current globalized and knowledge-based economies of rapidly changing post-industrialized societies.

Finally, we follow the suggestion of Toivonen et al. (2011) to leave out the Mertonian innovators from our model because they essentially constitute those individuals who illegally go outside legitimate means to accomplish cultural goals and therefore fall outside the scope of our interest, given that they do not constitute either marginalized or non-marginalized individuals. Thus, our model consists of the following classes of agents: (1) conformists, (2) deviants, (3) rebels, and (4) quiet mavericks. Furthermore, we consulted an expert on Merton's model to get a rough estimate on what the typical population proportions are for each class of agents in any given post-industrialized society (Toivonen, personal communication). Table 1 lists the population proportions recommended for our model:

**Table 1:** Population proportions and descriptions.

**Class**	**Population size (%)**	**Description**	**Marginalized?**
Rebel	4	Devises own legal means for accomplishing independently defined goals	No
Deviant	35	Deviates from mainstream goals, lacking the means to achieve them	Yes
Conformist	60	Embraces mainstream with access to the institutional means to accomplish them.	No
Quiet Maverick	1	Defines new goals that pave the way for future conformists and creates opportunities for deviants to become conformists	No

### 2.2. Background: macro-level factors

In trying to explain what macro-level factors play a role in increasing the population proportion of deviants in a society, Norasakkunkit et al. (2012) theorized that when access to institutional means that support cultural goals become constrained, the population proportion of conformists decreases while the population proportion of deviants increases. Also, in some cases the potential conformists with resources may choose to become rebels. The question is then, what constrains access to the institutional means that support cultural goals?

Norasakkunkit et al. (2012) argued that increased constraints on access are likely caused by higher degrees of resistance to changing institutional practices. But changes in practices are needed to adapt to changes in social and economic realities (e.g., increased global competition). Failure to adapt causes suboptimal performance. Therefore, higher degrees of resistance to change mean that institutions are performing at suboptimal levels. Inferior performance inhibits emergence of secure social spaces, which are needed for individuals to become conformists and gain access to the needed resources. One example of a costly resistance to change discussed by Norasakkunkit and colleagues is how Japanese private and public institutions are, for the most part, reluctant to switch from a seniority system to a meritocracy system, thereby creating generational inequality in the labor market that stifles fresh and innovative ideas. In such a scenario, there is increased potential for conformists to be pushed into the insecure social spaces of their society, thereby ending up as deviants (i.e., culturally marginalized individuals) who bear the brunt of the cost of the institutional disequilibrium due to high resistance to change.

In addition to resistance to change playing a role in modulating marginalization, we speculate that diversity plays an important role in the permeability of class boundaries. By “diversity,” we mean individual differences in traits and behaviors within each class of agents. If diversity is important, then we should see changes in population proportions of each class of agents. This argument stems from the work on cultural tightness and looseness by Gelfand et al. (2011). Cultural tightness and looseness refer to the degree to which a society has strong norms and a low tolerance of deviant behaviors. Relatively tight societies tend to have low tolerance for deviant behaviors and more severe sanctions against norm violations. Relatively loose societies tend to have a higher tolerance for deviance and are less likely to punish norm violations. Tightness inversely corresponds with diversity.

### 2.3. Hypotheses

Our hypotheses focus on two conditions: (1) low diversity and high resistance to change, and (2) high diversity and low resistance to change. Within these conditions, we are interested in three outcomes for each group: (A) population proportion, (B) cultural change, and (C) cultural similarity to Conformists. Our hypotheses for the groups for each of these conditions is presented in Table 2.

**Table 2:** Hypotheses.

	**Low diversity and high resistance to change**	**High diversity and low resistance to change**
Population proportion	Conformists: lowest Deviants: highest Rebels: highest Quiet Mavericks: no hypothesis	Conformists: highest Deviants: lowest Rebels: lowest Quiet Mavericks: no hypothesis
Cultural change	Conformists: highest Deviants: lowest Rebels: lowest Quiet Mavericks: no hypothesis	Conformists: lowest Deviants: highest Rebels: highest Quiet Mavericks: no hypothesis
Cultural similarity to Conformists	Deviants: lowest Rebels: lowest Quiet Mavericks: no hypothesis	Deviants: highest Rebels: highest Quiet Mavericks: no hypothesis

Our hypotheses come from Norasakkunkit et al. (2012) argument that higher degrees of resistance to change will be associated with increased population proportions of deviants and rebels, and decreased population proportions of conformists. Put another way, over time, increased resistance to change will tend to: (1) restrict the movement of agents across class boundaries from the deviant class to the conformist class; (2) increase the movement of agents across class boundaries in the opposite direction; and (3) somewhat increase the movement of agents across class boundaries from the conformist and deviant classes to the rebel class. The role of resistance to change on the population proportions of quiet mavericks were not discussed in Norasakkunkit and colleague's theorizing, so that relationship will remain exploratory in our current model.

Concerning Diversity, we believe that greater diversity translates into more looseness in society. Thus, we hypothesize that with greater diversity comes greater tolerance of norm violations within the mainstream conformists and therefore less of a reason to push somewhat culturally deviant conformists into the deviant class, and to a lesser extent, into the rebel class. In contrast, we speculate that lower diversity translates into greater cultural tightness in society. Thus, we hypothesize that with less diversity comes more severe sanctions for norm violations in the form of pushing somewhat culturally deviant conformists into the deviant class, and to a lesser extent, the rebel class.

These considerations suggest the following hypotheses for a timespan of 2.5 generations.^3^ Under conditions of low resistance to change and high diversity, there will be with the highest population proportion of conformists and the lowest population proportions of deviants and rebels, largely due to deviants and rebels crossing boundaries over to becoming conformists. Under conditions of high resistance to change and low diversity, there will be the lowest population proportion of conformists and highest population proportions of deviants and rebels, largely due to conformists crossing boundaries over to becoming deviants and rebels.

It is important to note here that we believe these predictions are more relevant to post-industrialized societies than to manufacturing or developing economies. This is because post-industrialized societies are where it can be especially costly for institutional practices to not keep up with social and economic changes and where tolerance of diversity in perspectives and thinking play right into the innovativeness and industriousness that can sustain a post-industrial economy at optimal levels (Markus and Conner, 2013).

## 3. Method and model description

To describe the model, we will use the ODD^4^ protocol (Grimm et al., 2006, 2010). The advantage of using the ODD protocol to report the model is that it offers a broad, systematic overview of the model's components as well as details concerning specific design concepts^5^. Following the suggestion of Janssen (2017), the model code and image of the user interface are available at *modelingcommons*.*org*/*browse*/*one*_*model*/5381. The modeling platform is NetLogo 5.2.0.

### 3.1. Overview

#### 3.1.1. Purpose

The purpose of the model is to explore the macro-level conditions for marginalization. We focus on three areas of change. First, we are interested in looking at shifts in the sizes of populations in the model. Table 1 (above) gives the initial set-up. Second, we are interested in looking at how similar or different subcultures become to one another over time. It is possible, for example, that the subcultures trend toward egalitarianism: many interactions end up with cultures more or less looking like each other. It is also possible that homophilic tendencies will override subcultural interactions and create greater distinctions among subcultures. Finally, we are interested in changes in cultures over time.

#### 3.1.2. Entities and state variables

##### 3.1.2.1. Entities: agents and classes

There are four classes of agents in our model: quiet mavericks, conformists, deviants, and rebels. In NetLogo, classes of agents are referred to as “breeds^6^”. We will use “breed” when describing the logic of the model and “subculture” when referring to the cultural realities that the model is modeling. Likewise, “resistance to change” and “diversity” (lowercase) will refer to cultural macro-conditions. “RTC” and “Diversity” (uppercase) will refer to the corresponding model variables. Each class represents one of the subcultures introduced in section 2.1, and every agent is assigned to a subculture.

##### 3.1.2.2. State variables

There are two types of variables in the model: agent variables (i.e., micro-level variables) and global variables (i.e., macro-level variables). We begin with agent variables.

Agents possess a set of traits: a string of numbers that identify them as members of a subculture and individuate them as members of that subculture. The traits that identify them as members of a culture are *primary traits*. An agent's primary traits are represented as a string of eight numbers. We chose that many primary traits because we wanted to ensure that the values for each subculture overlapped in specific ways and eight was the minimum number of values needed to achieve this. Agents also possess five, seven, or ten *secondary traits*. Secondary traits identify individuals within a culture. So while every conformist has the same set of primary traits, any pair of conformists may or may not overlap on secondary traits.

Primary traits for each breed of agent overlapped to some degree. The degrees of overlap are shown in Table 3. (The values for the primary traits associated with each breed is given in **Table 6**, where we discuss initializing the model.) For example, when the model is initialized, any quiet maverick and any conformist will share three primary traits; deviants and rebels will not share any primary traits. It is worth noting that even though deviants and rebels do not overlap on primary traits, they might overlap on secondary traits. This reflects the commonplace observation that people from different subcultures might be interested in the same things: conformists and deviants might have a shared enjoyment of baseball or gourmet cooking.

**Table 3:** Overlap among primary traits.

	**Quiet maverick**	**Conformist**	**Deviant**	**Rebel**
Rebel	2/8	1/8	0/8	8/8
Deviant	2/8	0/8	8/8	
Conformist	3/8	8/8		
Quiet Maverick	8/8			

The primary traits do double duty. On the one hand, they act as group variables by identifying and individuating classes: conformists (for example) are different from deviants because there is at least one primary trait associated with all conformists that isn't associated with any deviant. On the other hand, primary traits are agent variables, for agents are the bearers of these traits. But these dual roles for primary traits are two sides of the same coin. Primary traits are markers of membership in a class because they are crowd-sourced from the members of a particular culture. So for example all rebels begin with the primary traits {4 15 7 8 9 14 17 18}. After a few time steps, it may turn out that many agents who had this string of numbers as a part of their primary traits dropped “18” and picked up another value, say “37.” The new set of primary traits associated with rebels would be {4 15 7 8 9 14 17 37}. And 37 would now become part of the set of primary traits associated with rebels because a majority of rebels dropped “18” and picked up “37.” Details for this process are described below in the section 3.3.2.

While the primary traits identify the agent as a member of a breed, the *secondary traits* make individuals within the breed unique. The secondary traits are assigned randomly from a range beginning with “22” and ending with “50” (secondary traits begin at “22” because primary traits are drawn from a range starting with “0” and running to “21”). So while it's possible that two agents of a breed could be assigned the same secondary traits, it's extremely unlikely: in the Low Diversity condition (to be explained shortly), agents have five secondary traits, delivering a probability of 1 in 850,668 that any two agents have identical sets of secondary traits. in the High Diversity condition, the odds drop to 1 in 5.18 × 10^9^.

Another micro-level variable tracks lifespan and generation. Each agent has two variables tracking how many times they have interacted and what generation they are a part of. After 1,000 interactions, an agent “dies” and is replaced by another agent. We estimate an agent has roughly 1,000 interactions in a lifetime that can potentially change one's primary or secondary traits—roughly 14–20 a year for 50–70 years. Even if this number is off by a few hundred in either direction, it affects the model's running time (but not any of the interactions). The new agent is initially assigned to the same breed as the one that died, but the newly born agent doesn't take on the breed's traits wholesale. Rather, each new agent has the opportunity to take on all, some or none of the primary traits of its parent's culture. Our rationale here is that progeny don't necessarily take on all the cultural preferences of their parents. We let the platform's random number generator determine how similar offspring are to their parents. The model starts with generation 0 and ends with generation 2.5 (i.e., after generation 2 has, on average, 500 interactions)^7^. We integrate two macro-level variables into the model, introduced in section 2.2: resistance to change and diversity. RTC operationalizes resistance to change. It assigns probabilities for successful interactions. A successful interaction is one in which an agent adopts a trait of another agent, sometimes from across class boundaries. Suppose an arbitrarily chosen rebel picks out an arbitrarily chosen deviant to interact with; whether the rebel replaces one of its own primary or secondary traits with one of the deviant's is determined by a set of probabilities reflecting the likelihood that someone from the deviant's culture would successfully influence the rebel. These probabilities are reflected in the Table 4. The one doing the influencing is on the vertical and the one influenced is on the horizontal^8^.

**Table 4:** Resistance to change.

	**Quiet maverick**	**Conformist**	**Deviant**	**Rebel**
**PANEL A: LOW**				
Quiet Maverick	1.0	0.05	0.05	0.20
Conformist	0.0	1.0	0.10	0.0
Deviant	0.0	0.0	1.0	0.0
Rebel	0.0	0.10	0.60	1.0
**PANEL B: MODERATE**				
Quiet Maverick	1.0	0.0375	0.075	0.15
Conformist	0.0	1.0	0.075	0.0
Deviant	0.0	0.20	1.0	0.0
Rebel	0.0	0.075	0.40	1.0
**PANEL C: HIGH**				
Quiet Maverick	1.0	0.025	0.10	0.10
Conformist	0.0	1.0	0.05	0.0
Deviant	0.0	0.40	1.0	0.0
Rebel	0.0	0.05	0.20	1.0

Notice that agents of a class will always influence other agents of their same class and there are some classes for which agents never influence others: a quiet maverick will never take on values from anyone else; rebels will never take on values from a conformist.

RTC operationalizes resistance to change in the following way. As a society becomes more resistant to change, institutional practices become more entrenched and class boundaries become more difficult to breach. So a society's low resistance to change is captured in the model through a Low RTC relative to the other RTC conditions. There is one exception for the impermeability of class boundaries as resistance to change goes up: deviants are more likely to copy conformists (but the same doesn't hold of conformists copying deviants). This is because as conformists are more thoroughly entrenched and deviants have a more difficult time moving into other social spaces, their best option is to copy mainstream norms and values.

The macro-level condition of diversity reflects how diverse individuals within a subculture are. If, for example, an American subculture tolerates a great deal of variation, then there would be more ways to “do” that subculture. We capture diversity in the model, in terms of the number of secondary traits agents might have. The greater the number of secondary traits, the greater the possibility of variation and so the greater the level of Diversity. These levels are given in Table 5.

**Table 5:** Diversity of traits.

**Diversity**	**Primary traits**	**Secondary traits**	**Total traits**
Low	8	5	13
Medium	8	7	15
High	8	10	18

While we recognize that subcultures may have varying levels of diversity, we opted to reduce the degrees of complexity of the model and make subcultures in the model equally Diverse.

#### 3.1.3. Process overview and scheduling

The two macro-level variables are our two independent variables: RTC and Diversity. The result is a 3 × 3 design of High, Medium, and Low Diversity, and High, Medium, and Low RTC. There were 25 runs for each of the nine conditions, thereby resulting in a sample size of *N* = 9 × 25 = 225 cases for the purpose of hypotheses testing. Twenty five runs were done for each condition because between 15 and 30 trials is regarded as sufficient for generating reliable data (cf. Railsback and Grimm, 2012).

For each round of interactions between agents, the model recorded:

1. The population proportion for each class of agents (0.0 to 1.0);
2. Degree of within culture change from its starting point (0.0 to 1.0);
3. Degree of cultural similarity between any two breeds of agents, based on degree of similarity of traits (0.0 to 1.0).

As mentioned above, each run of the model lasted approximately 2.5 generations of agents and each generation lasts approximately 1,000 rounds of interactions. Population, cultural change, and similarity results were averaged over each run. These averaged values for pop, cultural change, and similarity over 2.5 generations are the three dependent variables in our analysis across the 9 conditions.

**Figure 1:**
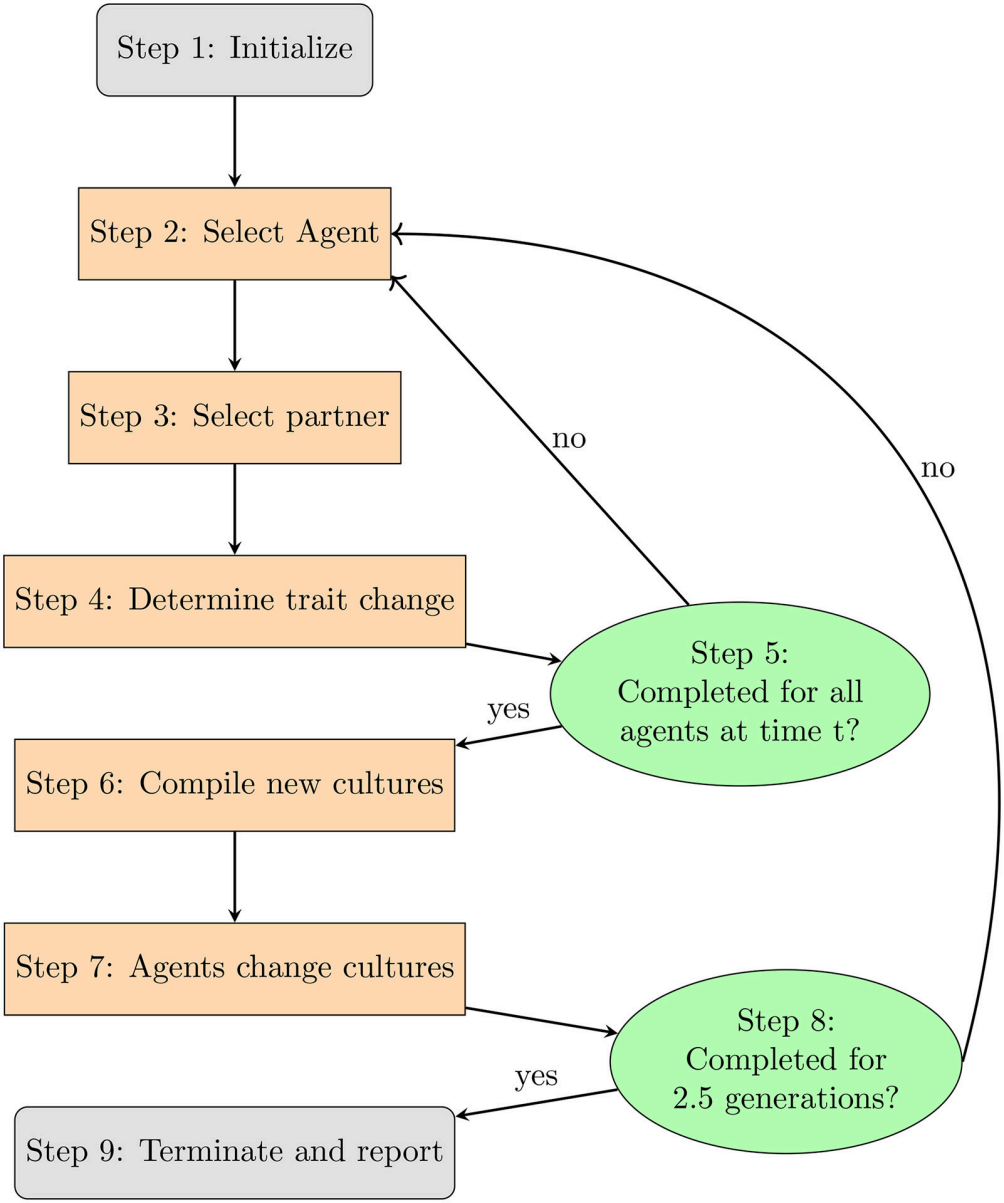
Model schematic.

Now with the relevant variables for exploration have been identified, we will turn to an overview of the model's flow. Figure 1 provides a schematic with each step explained in further detail below:^9^

1. The model is initialized with 200 agents distributed among subcultures according to the proportions given in Table 1 and assigned primary and secondary traits.
2. An agent $A_{B}^{i}$ is selected at random. The subscript is a variable indicating the agent's breed. The superscript takes a value 0 ≤ *i* ≤ n, where *n* is the total number of agents that have existed in the model.^10^ The value *i* uniquely identifies an agent.
3. $A_{B}^{i}$ selects another agent $A_{B}^{p}$ (where *i* ≠ *p*) with whom to interact.
4. Table 4 lists the likelihood that $A_{B}^{i}$ replaces one of its own primary or secondary traits with one belonging to $A_{B}^{p}$. If the platform's random number generator selects a real number that is below the threshold for interaction give the breeds for $A_{B}^{i}$ and $A_{B}^{p}$, then $A_{B}^{i}$ adopts a trait from $A_{B}^{p}$. Procedures for interactions are sketched out in section 3.2.2 and detailed in section 3.3.2.)
5. Steps 2–4 are repeated for each agent.
6. Some agents have different values for their primary and secondary traits. For each class of agents, the program compiles all agents' primary and secondary traits into a single list. It identifies the eight most frequently occurring values and defines that list as the new primary traits for that breed. In cases of ties, the program chooses a value at random.
7. Once new cultures have been compiled, each $A_{B}^{i}$ checks to see which culture best matches its own list of primary and secondary traits. If the agent's list of primary and secondary traits contains the same 8 values as some culture, then that agent becomes a member of that culture (ties are determined by a coin-flip).
8. Agents then check how many interactions they have undergone. If the number exceeds 1,000, the agent dies and is replaced by another agent that has up to 8 of the initial agent's traits. These values populate the newly-born agent's list of primary traits. The remaining traits are determined at random.
9. Finally, the model reports the extent of a culture's changes over time, similarities to other cultures, as well as shifts in the population.

### 3.2. Design concepts

Grimm et al. (2010) identify 11 design concepts. Here, we address those that are pertinent to our model.

#### 3.2.1. Basic principles

The micro- and macro-level properties that are being modeled have been discussed in section 2. And the dependent variables were identified in section 3.1.3. We're interested in examining the effects of RTC and Diversity on population of each breed of agents, the degree of change within a subculture, and the degree of similarity between two subcultures.

The inspiration for the model is Axelrod (1997). In that model, values for cultures are assigned randomly as a string of five, ten, or fifteen digits (depending on the trial). Each digit's “slot” in the string could take a value from 0 to five, ten, or fifteen (again, depending on the trial). This resulted in a 3 × 3 design. Each culture (represented as a spatial patch on a 10 × 10 grid) would pick one of its neighbors as a partner. Cultures would interact with a probability equal to their similarity. The culture seeking out a neighboring culture with which to interact replaces one of its own values with one from the neighbor. Suppose patch *P*_1_ has a neighbor *P*_2_ and they share three of five values. The likelihood of their interacting, and *P*_1_ adopting a value of *P*_2_ is 60%. One of Axelrod's questions is: how many stable regions emerge under each of these conditions?

On average, one stable region developed for most conditions^11^. A notable exception is the condition in which there were five cultural features and fifteen traits per feature. Under this condition, there were on average 20 stable regions. One of the interesting findings of Axelrod's paper is that the number of stable regions increases nonlinearly when there are few features but many traits per feature.

We are impressed with the degree of convergence shown in Axelrod's model, and also with the exponential increase of stable regions under conditions of low features and many traits. We have followed Axelrod in broad strokes, letting randomness control much of the outcomes of the model (more on this in section 3.2.3). We follow Axelrod in his modeling choice to leave much up to chance for the same reasons. Social interaction is a complex affair with countless many forces shaping our decision. The use of probabilities leaves to the fates how things will turn out for agents in the model without our having to micro-manage their interactions.

#### 3.2.2. Interaction

Every agent interacts with one other agent at each timestep. We envision this interaction as follows: the initiating agent $A_{B}^{i}$ selects one value *v* from its list of primary and secondary traits. It then finds a partner $A_{B^{\prime }}^{p}$ who also has *v* in its list of traits. (B and B′ may be the same breed.) $A_{B}^{i}$ then selects a floating point number *n* between 0 and 1.0; if *n* is below the threshold needed to have a successful interaction (see Table 4), then $A_{B}^{i}$ replaces one of its traits with a randomly selected trait from $A_{B}^{p}$. If *n* doesn't pass the threshold, then neither $A_{B}^{i}$ nor $A_{B}^{p}$ changes as a result of the interaction.

#### 3.2.3. Stochasticity

Stochasticity plays an important role in our model. First, the secondary traits of each agent are determined by NetLogo's random number generator. Second, whether an agent passes the threshold for successfully interacting with another agent depends on the same number generator. Finally, the degree of similarity between a newly born agent and its deceased progenitor is determined randomly: as mentioned in sections 3.1.2 and 3.1.3, offspring can take on all, some or none of their parents' cultural values and the remaining values are set at random.

Our model incorporates stochasticity at many levels because there are many forces that affect how traits get passed on both within interactions and across generations. It is implausible to suppose that offspring are exactly like their parents. It's also implausible to suppose people are influenced by others every time they interact. So to capture the turbulence of social interactions, we rely on a random number generator to determine when agents successfully influence others.

#### 3.2.4. Collectives

Every agent belongs to a culture: quiet mavericks, conformists, rebels, or deviants. Culture membership determines an agent's primary traits and also the probabilities for successful interactions.

### 3.3. Details

#### 3.3.1. Initialization

There are 200 agents, and they are assigned to one of four cultures with the primary traits listed in Table 6:

**Table 6:** Initialization of agents.

**Culture**	**Number**	**Primary traits**
Conformist	120	{ 1 2 3 4 5 16 19 20}
Quiet Maverick	2	{1 2 3 7 10 13 17 18}
Rebels	8	{4 15 7 8 9 14 17 18}
Deviant	70	{6 8 9 11 12 13 21 22}

#### 3.3.2. Submodels

Let:

- *A*_*B*_ indicate the set of all agents of a breed *B*.
- $A_{B}^{i}$ indicate an arbitrarily chosen agent *i* of breed *B*. The superscript *i* will be replaced with *p* when identifying the interaction partner for $A_{B}^{i}$. Each agent has a unique, identifying value for *i*. Note that *A*_*B*_ = $\cup A_{B}^{i}$ for all *i* of a breed *B*. *A* = ∪*A*_*B*_ for all breeds *B*.
- $C_{P}{\left(A_{B}^{i}\right)}_{t}$ indicate the primary traits of agent *i* who is a member of breed *B* at time *t*. $C_{S}{\left(A_{B}^{i}\right)}_{t}$ indicates the secondary traits of agent *i* at *t*. And $C{\left(A_{B}^{i}\right)}_{t}$ = $C_{P}{\left(A_{B}^{i}\right)}_{t}\cup C_{S}{\left(A_{B}^{i}\right)}_{t}$
- *e*_*n*_ be an arbitrary element of $C{\left(A_{B}^{i}\right)}_{t}$, indicated $e_{n}\in C{\left(A_{B}^{i}\right)}_{t}$

##### 3.3.2.1. Interaction

This submodel has been sketched above. Here we fill out the details. From *A*, the submodel selects some $A_{B}^{i}$ and identifies $e_{n}\in C{\left(A_{B}^{i}\right)}_{t}$. Then the submodel searches *A* for some $A_{B^{\prime }}^{p}$ for which $e_{n}\in C{\left(A_{B}^{p}\right)}_{t}$ (where *i*≠*p*), and it may or may not be the case that *B* = *B*′. If there is some $A_{B}^{p}$, then a random floating point number *r* is selected. If *r* is below the threshold for interaction—which is determined by the level of resistance chosen at initialization and by the breeds of $A_{B}^{i}$ and $A_{B}^{p}$ (specified in Table 4)—then some *e*_*m*_ such that $e_{m}\in C{\left(A_{B}^{i}\right)}_{t}$ is replaced with some *e*_*m*_ such that $e_{m^{\prime }}\in C{\left(A_{B}^{p}\right)}_{t}$. As a result of the interaction $e_{m^{\prime }}\in C{\left(A_{B}^{i}\right)}_{t}$ as well as $e_{m^{\prime }}\in C{\left(A_{B}^{p}\right)}_{t}$, that is $\exists e\left(e\in \left\{C{\left(A_{B}^{i}\right)}_{t}\cap C{\left(A_{B}^{p}\right)}_{t}\right)\right\}$.

##### 3.3.2.2. Analyzing cultures

This submodel discovers the most common values found among all agents of a breed and then identifies a new set of eight primary traits that define the breed. It first compiles *T* = $\{e_{n}|e_{n}\in \left(C{\left(A_{B}^{i}\right)}_{t}\right\}$ for each ${\left(A_{B}^{i}\right)}_{t}\in A_{B}$. Then the submodel identifies the eight most frequently occurring values in *T*. The resulting list a new *C*(_*A*_*B*_)*t*+1_, is the updated list of primary traits for membership in *A*_*B*_. This submodel runs at every time step. For any times *t* and *t* + *n*, it is sometimes the case that *C*(_*A*_*B*_)*t*_ = *C*(*A*_*B*_)_*t*+*n*_.

##### 3.3.2.3. Switching breeds

Each $e_{n}\in C{\left(A_{B}^{i}\right)}_{t}$ is compared against each *e*_*n*_∈*C*(*A*_*B*_) for each breed. If more than seven $e_{n}\in C{\left(A_{B}^{i}\right)}_{t}$ show up in *C*(_*A*_*B*_)*t*_ for a breed *B*, then $A_{B}^{i}$ becomes a member of the breed *B*. Sometimes the agent's breed at time *t*+ 1 will be the same as its breed at *t*.

Our rationale for requiring agents' primary and secondary traits to match on all eight values of a breed's culture instead of requiring (say) a simple majority is that people can change in many ways and yet still be identified as a member of that culture. This is part of how cultures evolve over time: members make a number of small changes, which contribute to the evolution of the subculture, until they are recognizably a part of a different culture.

##### 3.3.2.4. New generations

Each $A_{B}^{i}$ has two variables: one tracks the number of interactions an agent has had and the other tracks the agent's generation. After 1,000 interactions, $A_{B}^{i}$ dies and is replaced by another agent $A_{B}^{o}$ of the same culture (*o* for ‘offspring'). Though $A_{B}^{o}$ is of the same breed as $A_{B}^{i}$, $C{\left(A_{B}^{o}\right)}_{t}$ is populated by $e_{1}\ldots e_{n}\in C{\left(A_{B}^{i}\right)}_{t}$ where *n* ≤ 8, and the value of *n* is determined randomly. And the remaining values for $C{\left(A_{B}^{o}\right)}_{t}$ are also chosen randomly.

## 4. Results

We said in section 3.1.3 that we tracked population proportions, cultural change, and similarity across culture. To test our above stated hypotheses with respect to changes in population of different classes of agents, an RTC(3) by Diversity(3) ANOVA was performed on population of deviants, population of conformists, and population of rebels. Results for population of quiet mavericks will be examined for exploratory purposes only.

In addition to testing our hypotheses with respect to the population variables, the RTC by Diversity ANOVA was also performed on cultural change and similarity to test the hypotheses that the higher the RTC and/or the higher the Diversity level(s), (1) the greater the degree of cultural change among conformists, and (2) the greater that change will occur to become more similar to deviants and rebels than the other way around. Here too, results for similarity to quiet mavericks will be examined for exploratory purposes only.

### 4.1. Population proportions

**Figure 2:**
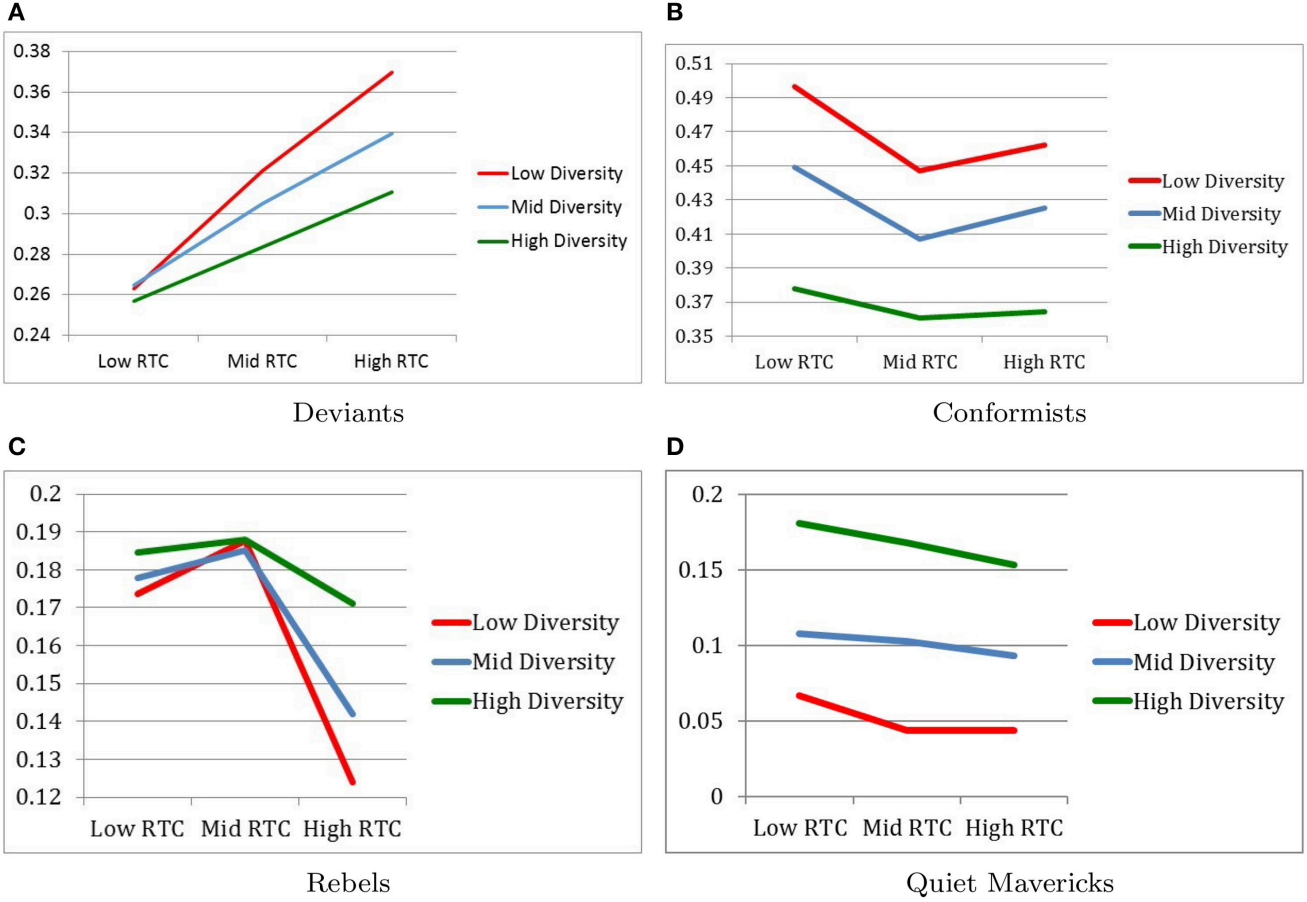
Changes in population proportions. **(A)** Deviants. **(B)** Conformists. **(C)** Rebels. **(D)** Quiet mavericks.

Figure 2 describes changes in population proportions among the subcultures.

#### 4.1.1. Population proportions of deviants

As expected, the population of deviants was smallest in the Low RTC condition (*M* = 0.2615, *SD* = 0.0211) and largest in the High RTC condition (*M* = 0.3400, *SD* = 0.0339) with the Moderate RTC condition (*M* = 0.3032, *SD* = 0.0254) falling in between, as indicated by *F*_(2, 216)_ = 243.32, *p* < 0.001, η^2^ = 0.693. *Post-hoc* pairwise comparisons confirm that all three RTC conditions were significantly different from each other with respect to the population proportions of deviants.

Also as expected, the population of deviants was smallest in the High Diversity condition (*M* = 0.2836, *SD* = 0.0279) and largest in the Low Diversity condition (*M* = 0.3180, *SD* = 0.0507) with Moderate Diversity condition (*M* = 0.3031, *SD* = 0.0377) falling in between, as indicated by *F*_(2, 216)_ = 46.9, *p* < 0.001, η^2^ = 0.303. *Post-hoc* pairwise comparisons confirm that all three Diversity conditions were significantly different from each other with respect to the population proportions of deviants.

Finally, the interaction between RTC and Diversity confirmed the expectation that population of deviants was largest when RTC was high and Diversity was low, while the population of deviants was smallest when RTC was low and Diversity was high, as indicated by *F*_(4, 216)_ = 9.5, *p* < 0.001, η^2^ = 0.150 (see Figure 2A).

#### 4.1.2. Population proportion of conformists

As expected, the population of conformists was largest in the Low RTC condition (*M* = 0.4409, *SD* = 0.0597) relative to the other RTC conditions. However, contrary to expectations, the population of conformists was smallest in the Moderate RTC condition (*M* = 0.4049, *SD* = 0.0405) with the High RTC condition (*M* = 0.4173, *SD* = 0.0471) falling in between, as indicated by *F*_(2, 216)_ = 35.76, *p* < 0.001, η^2^ = 0.249. *Post-hoc* pairwise comparisons confirm that all three RTC conditions were significantly different from each other with respect to the population proportions of conformists.

Also as expected, the population of conformists was smallest in the High Diversity condition (*M* = 0.3676, *SD* = 0.0251) and largest in the Low Diversity condition (*M* = 0.4684, *SD* = 0.0329) with the Moderate Diversity condition (*M* = 0.4271, *SD* = 0.0335) falling in between, as indicated by *F*_(2, 216)_ = 273.85, *p* < 0.001, η^2^ = 0.717. *Post-hoc* pairwise comparisons confirm that all three Diversity conditions were significantly different from each other with respect to the population proportions of conformists.

Finally, the interaction between RTC and Diversity confirmed the expectation that population of conformists was largest when RTC was low and Diversity was low, as indicated by *F*_(4, 216)_ = 2.75, *p* < 0.05, η^2^ = 0.048. As expected also, population of conformists was small when Diversity was high, but no difference was found between moderate and high RTC conditions (see Figure 2B).

#### 4.1.3. Population proportion of rebels

We anticipated that rebels would follow the same patterns as deviants. But contrary to our expectations the population of rebels was smallest in the High RTC condition (*M* = 0.1457, *SD* = 0.0440) rather than in the Low RTC condition (*M* = 0.1786, *SD* = 0.0249), as indicated by *F*_(2, 216)_ = 36.89, *p* < 0.001, η^2^ = 0.255. Furthermore, according to *Post-hoc* pairwise comparisons, no statistical difference was found between the Low RTC condition and the Moderate RTC condition (*M* = 0.1868, *SD* = 0.0257), and the High RTC condition was statistically different from both the Moderate and Low RTC conditions.

Unlike deviants, the population of rebels was largest in the High Diversity condition (*M* = 0.1812, *SD* = 0.0261) rather than in the Low Diversity condition (*M* = 0.1617, *SD* = 0.0449), as indicated by *F*_(2, 216)_ = 7.63, *p* = 0.001, = 0.066. Furthermore, according to *Post-hoc* pairwise comparisons, no statistical difference was found between the Low Diversity condition and the Moderate Diversity condition (*M* = 0.1682, *SD* = 0.0357), and the High Diversity condition was statistically different from both the Moderate and Low Diversity conditions.

Finally, the interaction between RTC and Diversity contradicted our expectations, which was the same as that for population for deviants. Specifically, it was found that the population of rebels was smallest when RTC was high and Diversity was low instead of in the opposite conditions, as indicated by *F*_(4, 216)_ = 4.00, *p* < 0.01, η^2^ = 0.069 (see Figure 2C).

#### 4.1.4. Population proportion of quiet mavericks

The results for the population of quiet mavericks were exploratory since we did not have any hypotheses regarding these results. Analysis shows that there were only main effects of RTC and Diversity but no interaction effect between RTC and Diversity.

The main effect of RTC suggested that the population of quiet mavericks were larger in the Low RTC condition (*M* = 0.1184, *SD* = 0.0598) than in the Moderate RTC condition (*M* = 0.1045, *SD* = 0.0571) and the High RTC condition (*M* = 0.0964, *SD* = 0.0542), as indicated by *F*_(2, 216)_ = 9.34, *p* < 0.001, η^2^ = 0.08. However, *post-hoc* pairwise comparisons indicated that there was no statistically significant difference between the high RTC condition and the Moderate RTC condition with respect to the population proportions of quiet mavericks.

The main effect of Diversity suggested, the population of quiet mavericks was largest in the High Diversity condition (*M* = 0.1670, *SD* = 0.0298) and smallest in the Low Diversity condition (*M* = 0.0512, *SD* = 0.0304) with Moderate Diversity condition (*M* = 0.1010, *SD* = 0.0372) falling in between, as indicated by *F*_(2, 216)_ = 254.833, *p* < 0.001, η^2^ = 0.702. *Post-hoc* pairwise comparisons confirm that all three Diversity conditions were significantly different from each other with respect to the population proportions of quiet mavericks. It is also notable that the effect size of Diversity on population of quiet mavericks was very large (see Figure 2D).

### 4.2. Cultural changes over 2.5 generations

**Figure 3:**
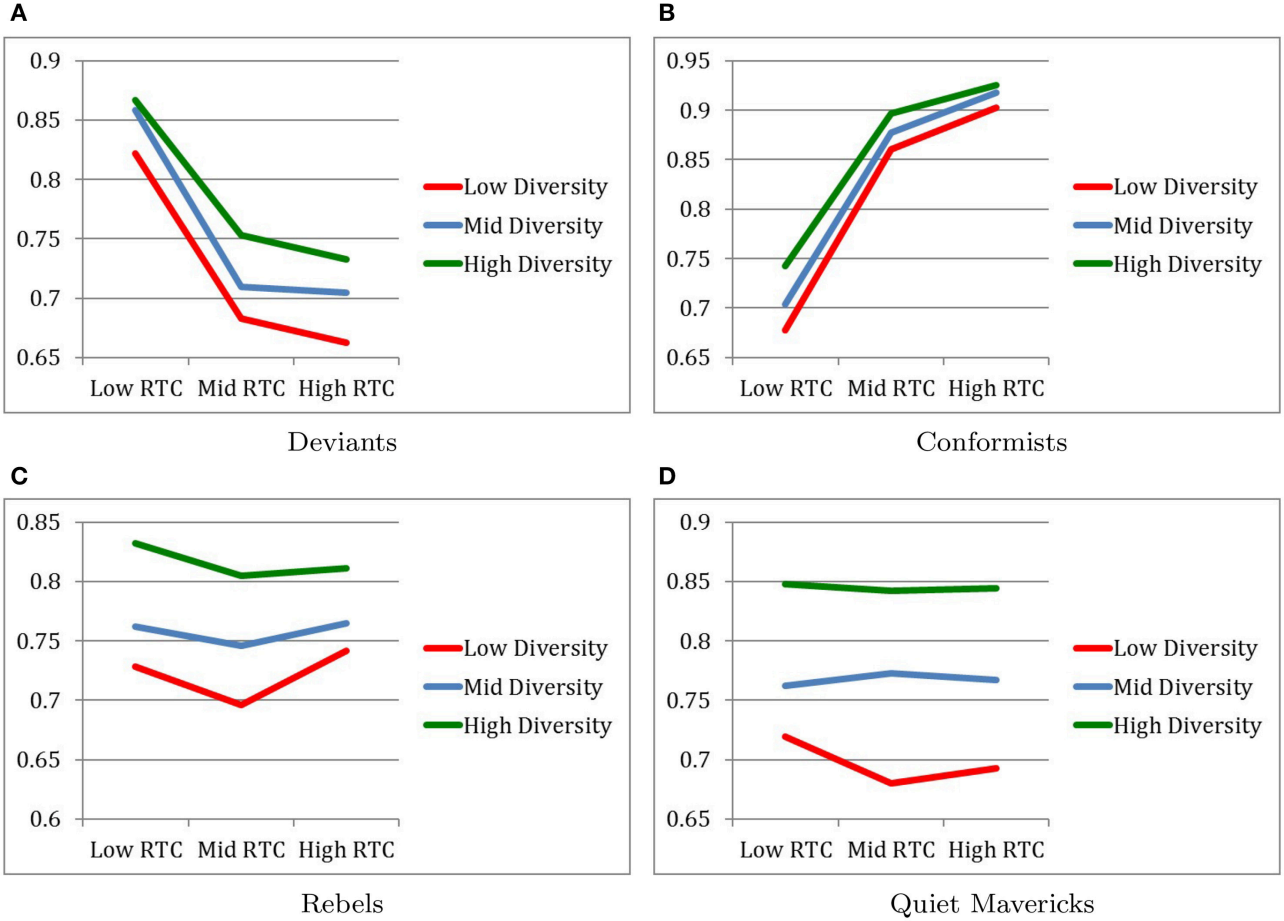
Degrees of cultural change over 2.5 generations. **(A)** Deviants. **(B)** Conformists. **(C)** Rebels. **(D)** Quiet mavericks.

Figure 3 describes changes in the subcultural traits over 2.5 generations.

#### 4.2.1. Cultural change of deviants

As expected, the main effect of RTC revealed that cultural change for deviants occurred to the least degree under the High RTC condition (*M* = 0.7000, *SD* = 0.0594) and occurred to the greatest degree in the Low RTC condition (*M* = 0.8669, *SD* = 0.0239) with the moderate RTC condition (*M* = 0.7151, *SD* = 0.0596) falling in between, as indicated by *F*_(2, 216)_ = 225.43, *p* < 0.001, η^2^ = 0.676.

*Post-hoc* pairwise comparisons confirm that all three RTC conditions were significantly different from each other with respect to degree of cultural change of deviants.

Also as expected, the main effect of Diversity revealed that cultural change for deviants occurred to the greatest degree under the High Diversity condition (*M* = 0.7841, *SD* = 0.0882) and occurred to the least degree in the Low Diversity condition (*M* = 0.7224, *SD* = 0.0882) with the Moderate Diversity condition (*M* = 0.7576, *SD* = 0.0861) falling in between, as indicated by *F*_(2, 216)_ = 32.22, *p* < 0.001, η^2^ = 0.23. *Post-hoc* pairwise comparisons confirm that all three Diversity conditions were significantly different from each other with respect to the degree of cultural change of conformists.

No interaction effect between RTC and Diversity on the degree of cultural change for deviants was found (see Figure 3A).

#### 4.2.2. Cultural change of conformists

As expected, the conformists were more likely to experience cultural change over 2.5 generations under relatively higher RTC and relatively higher diversity conditions.

Specifically, as expected, the main effect of RTC revealed that cultural change for conformists occurred to the greatest degree under the High RTC condition (*M* = 0.9150, *SD* = 0.0241) and occurred to the least degree in the Low RTC condition (*M* = 0.7079, *SD* = 0.0564) with the Moderate RTC condition (*M* = 0.8780, *SD* = 0.0338) falling in between, as indicated by *F*_(2, 216)_ = 689.14, *p* < 0.001, η^2^ = 0.865. *Post-hoc* pairwise comparisons confirm that all three RTC conditions were significantly different from each other with respect to degree of cultural change of conformists. It is also notable that the effect size of RTC on cultural change for conformist was very large.

Also as expected, the main effect of Diversity revealed that cultural change for conformists occurred to the greatest degree under the High Diversity condition (*M* = 0.8545, *SD* = 0.0876) and occurred to the least degree in the Low Diversity condition (*M* = 0.8132, *SD* = 0.1064) with the Moderate Diversity condition (*M* = 0.8332, *SD* = 0.0988) falling in between, as indicated by *F*_(2, 216)_ = 24.02, *p* < 0.001, η^2^ = 0.182. *Post-hoc* pairwise comparisons confirm that all three Diversity conditions were significantly different from each other with respect to the degree of cultural change of conformists.

The interaction between RTC and Diversity revealed a marginally significant trend that cultural change for conformists occurred to the greatest degree in the combination of High RTC and High Diversity, as indicated by *F*_(4, 216)_ = 2.31, *p* < 0.059, η^2^ = 0.041 (see Figure 3B).

#### 4.2.3. Cultural change of rebels

While cultural change for rebels changed in the expected direction as a function of Diversity, cultural change for rebels as a function of RTC was unexpected.

Specifically, the main effect of RTC revealed that cultural change for rebels occurred to the greatest degree under the Moderate RTC condition (*M* = 0.7489, *SD* = 0.0745) relative to the Low RTC condition (*M* = 0.7742, *SD* = 0.0708) and the High RTC condition (*M* = 0.7725, *SD* = 0.0602), as indicated by *F*_(2, 216)_ = 4.64, *p* = 0.011, η^2^ = 0.04. *Post-hoc* pairwise comparisons reveal that there was no statistically significant difference between the High and Low RTC conditions. It is also notable that the effect size of RTC on cultural change for rebels was small.

However, as expected, the main effect of Diversity revealed that cultural change for rebels occurred to the greatest degree under the High Diversity condition (*M* = 0.8159, *SD* = 0.0460) and occurred to the least degree in the Low Diversity condition (*M* = 0.7221, *SD* = 0.0719) with the Moderate Diversity condition (*M* = 0.7575, *SD* = 0.0525) falling in between, as indicated by *F*_(2, 216)_ = 58.88, *p* < 0.001, η^2^ = 0.402. *Post-hoc* pairwise comparisons confirm that all three Diversity conditions were significantly different from each other with respect to the degree of cultural change of rebels.

No interaction effect between RTC and Diversity on the degree of cultural change for rebels was found (see Figure 3C).

#### 4.2.4. Cultural change of quiet mavericks

The degree of cultural change for quiet mavericks was examined for exploratory purposes.

No main effect of RTC on degree of cultural change for quiet mavericks was found. However, not surprisingly, the main effect of Diversity revealed that cultural change for quiet mavericks occurred to the greatest degree under the High Diversity condition (*M* = 0.8441, *SD* = 0.0486) and occurred to the least degree in the Low Diversity condition (*M* = 0.6927, *SD* = 0.0690) with the Moderate Diversity condition (*M* = 0.7667, *SD* = 0.0613) falling in between, as indicated by *F*_(2, 216)_ = 119.64, *p* < 0.001, η^2^ = 0.526. *Post-hoc* pairwise comparisons confirm that all three Diversity conditions were significantly different from each other with respect to the degree of cultural change of quiet mavericks.

No interaction effect between RTC and Diversity on the degree of cultural change for quiet mavericks was found (see Figure 3D).

### 4.3. Similarity of other subcultures to conformists

**Figure 4:**
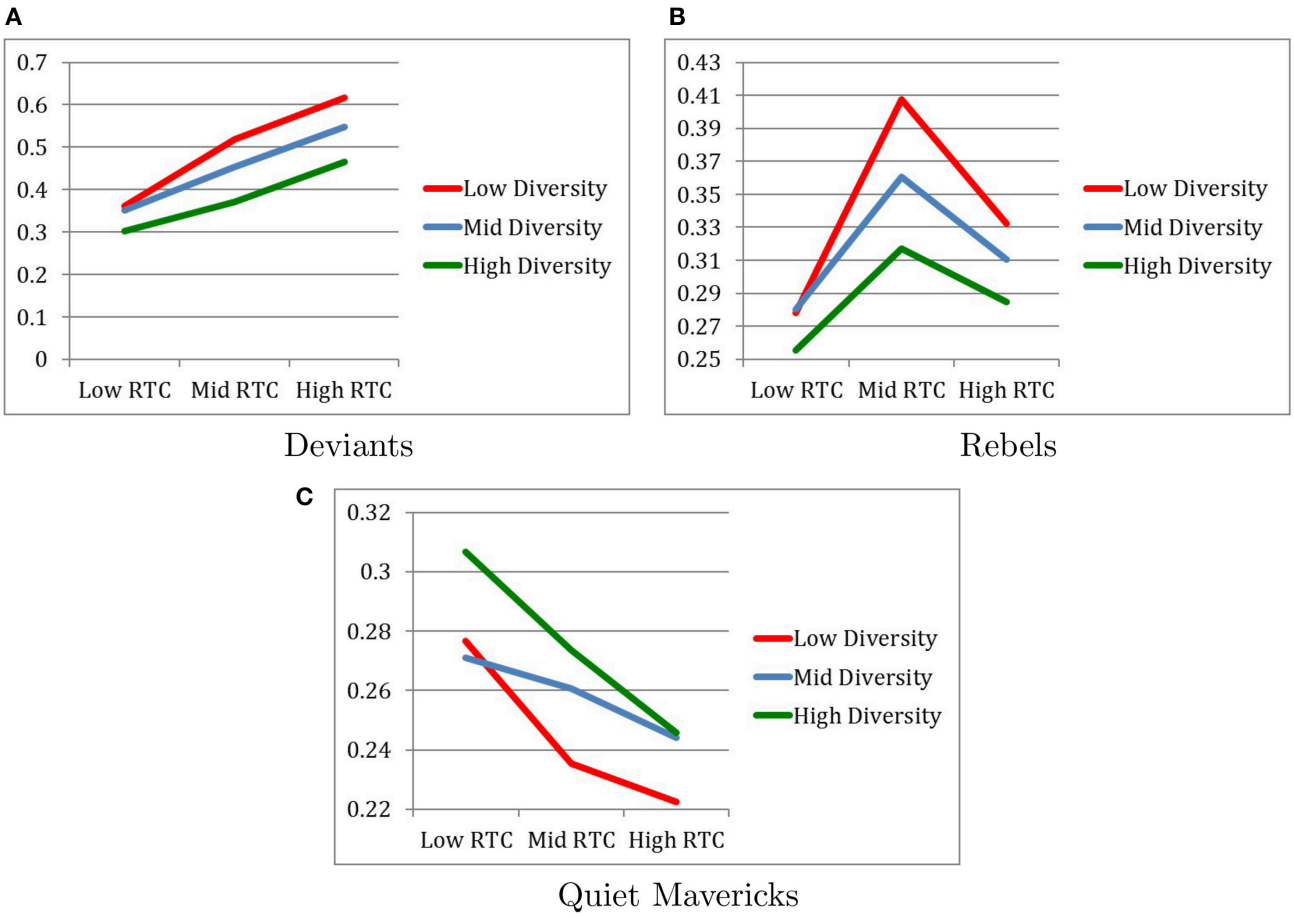
Similarity to conformists after 2.5 generations. **(A)** Deviants. **(B)** Rebels. **(C)** Quiet mavericks.

Figure 4 describes changes in the degree of similarity between conformists and the other three groups.

#### 4.3.1. Similarity between conformists and deviants

When the similarity of conformists and deviants over 2.5 generations was examined, as expected, the higher the RTC, the more conformists changed in the direction of becoming more similar to deviants than the other way around. That is, the direction of similarity between conformists and deviants was in the same direction as the cultural change for conformists but opposite of the direction of the cultural change for deviants.

Specifically, as expected, the main effect of RTC revealed that conformists and deviants most similar under the High RTC condition (*M* = 0.5430, *SD* = 0.0645) and least similar under Low RTC condition (*M* = 0.3386, *SD* = 0.0391) with the Moderate RTC condition (*M* = 0.4473, *SD* = 0.0688) falling in between, as indicated by *F*_(2, 216)_ = 825.34, *p* < 0.001, η^2^ = 0.884. *Post-hoc* pairwise comparisons confirm that all three RTC conditions were significantly different from each other with respect to degree of similarity between conformists and deviants. It is also notable that the effect size of RTC on degree of similarity between conformist and deviants was very large.

Contrary to our expectation, the main effect of Diversity revealed that conformists and deviants were most similar under the Low Diversity condition (*M* = 0.4984, *SD* = 0.1094) and least similar under the High Diversity condition (*M* = 0.3796, *SD* = 0.0748) with the Moderate Diversity condition (*M* = 0.4509, *SD* = 0.0857) falling in between, as indicated by *F*_(2, 216)_ = 282.31, *p* < 0.001, η^2^ = 0.723. *Post-hoc* pairwise comparisons confirm that all three Diversity conditions were significantly different from each other with respect to the degree of similarity between conformists and deviants. It is also notable that the effect size of Diversity on degree of similarity between conformist and deviants was very large.

The interaction between RTC and Diversity revealed a marginally significant trend that conformists and deviants were most similar in the combination of High RTC and Low Diversity, as indicated by *F*_(4, 216)_ = 17.92, *p* < 0.059, η^2^ = 0.249 (see Figure 4A).

#### 4.3.2. Similarity between conformists and rebels

When the similarity of conformists and rebels over 2.5 generations was examined, the hypothesis that the higher the RTC, the more similar conformists and rebels are, was partially supported. Specifically, conformists and rebels were most similar in the Moderate RTC condition (*M* = 0.3618, *SD* = 0.0537) rather than the High RTC condition (*M* = 0.3092, *SD* = 0.0603), but as expected, they were the least similar in the Low RTC condition (*M* = 0.2713, *SD* = 0.0440), as indicated by *F*_(2, 216)_ = 68.90, *p* < 0.001, η^2^ = 0.389. *Post-hoc* pairwise comparisons confirm that all three RTC conditions were significantly different from each other with respect to degree of similarity between conformists and rebels.

Contrary to our expectation, the main effect of Diversity revealed that conformists and rebels were most similar under the Low Diversity condition (*M* = 0.3393, *SD* = 0.0723) and least similar under the High Diversity condition (*M* = 0.2859, *SD* = 0.0452) with the Moderate Diversity condition (*M* = 0.3170, *SD* = 0.0627) falling in between, as indicated by *F*_(2, 216)_ = 23.97, *p* < 0.001, η^2^ = 0.182. *Post-hoc* pairwise comparisons confirm that all three Diversity conditions were significantly different from each other with respect to the degree of similarity between conformists and rebels.

The interaction between RTC and Diversity revealed a significant effect in which conformists and deviants were most similar in the combination of Moderate RTC and Low Diversity, as indicated by *F*_(4, 216)_ = 3.49, *p* < 0.01, η^2^ = 0.061, although this effect was small (see Figure 4B).

#### 4.3.3. Similarity between conformists and quiet mavericks

When the similarity of conformists and quiet mavericks over 2.5 generations was examined for exploratory purposes, conformists and quiet mavericks were most similar in the Low RTC condition (*M* = 0.2847, *SD* = 0.0635) and least similar in the High RTC condition (*M* = 0.2375, *SD* = 0.0470), with the Moderate RTC condition (*M* = 0.2565, *SD* = 0.0450) falling in between, as indicated by *F*_(2, 216)_ = 16.16, *p* < 0.001, η^2^ = 0.13. *Post-hoc* pairwise comparisons confirm that all three RTC conditions were significantly different from each other with respect to degree of similarity between conformists and quiet mavericks.

The main effect of Diversity revealed that conformists and quiet mavericks were more similar under the High Diversity condition (*M* = 0.2753, *SD* = 0.0561) relative to the Low Diversity condition (*M* = 0.2448, *SD* = 0.0553) and the Moderate Diversity condition (*M* = 0.2585, *SD* = 0.0523), as indicated by *F*_(2, 216)_ = 6.68, *p* < 0.001, η^2^ = 0.058. *Post-hoc* pairwise comparisons revealed that there was no statistically significant difference between the Low and Moderate Diversity conditions with respect to the degree of similarity between conformists and quiet mavericks.

No interaction effect between RTC and Diversity on the degree of similarity between conformists and quiet mavericks was found (see Figure 4C).

## 5. Discussion

In the current agent-based-modeling study, the hypotheses regarding population proportions of conformists and deviants as well as how they relate to each other and change over time as a function of RTC and Diversity levels were largely confirmed. The hypotheses regarding rebels were either partially confirmed or not confirmed. It is important to state here that we were more confident in our hypotheses regarding the conformists and deviants because they were more empirically grounded (see Norasakkunkit and Uchida, 2011, 2014; Uchida and Norasakkunkit, 2015). In contrast, there was little to no empirical foundation for hypothesizing what would happen to rebels as a function of RTC and Diversity levels, although we had some logical bases for those hypotheses.

Table 7 contains a summary of our main findings.

**Table 7:** Summary of main findings.

**Subculture**	**RTC**	**Diversity**	**Interaction?**
**(A) CONDITIONS FOR GREATEST POPULATION GROWTH**			
Conformist	Low	High	Yes
Deviant	High	Low	Yes
Rebel	Low	High	Yes
Quiet Mavericks	Low	High	No
**(B) CONDITION FOR GREATEST DEGREE OF CULTURAL CHANGE**			
Conformist	High	High	Yes
Deviant	Low	High	No
Rebel	Moderate	High	No
Quiet Mavericks	No effect	High	No
**(C) CONDITIONS FOR GREATEST DEGREE OF SIMILARITY TO CONFORMISTS**			
Deviant	High	Low	Yes
Rebel	Moderate	Low	Yes
Quiet Mavericks	Low	High	No

### 5.1. Deviants and conformists

Overall, our findings with respect to deviants confirmed that *as RTC levels increased*, their population proportion increased and their capacity to cross over class boundaries decreased. Furthermore, as RTC levels increased, deviants were less likely to undergo cultural change by being influenced by other classes of agents. The lack of diversity also had the expected effect on deviants as well (similar to increased RTC levels). That is, *as diversity levels decreased*, the population proportion of deviants increased and their capacity to cross over class boundaries decreased. Furthermore, as diversity levels decreased, deviants were less likely to undergo cultural change by being influenced by other classes of agents. RTC and Diversity also interacted with respect to population proportion of deviants. That is, the population proportion of deviants was largest in the combination of high RTC and low diversity. These patterns for deviants make sense in light of the idea that high RTC conditions keep deviants in their places, occupying marginalized social spaces of their society. High RTC also provides little opportunity for deviants to change from that state, especially when diversity in their class is low.

The patterns for conformists were also more or less expected. Specifically, the population proportions of conformists were higher under the low RTC condition relative to the other RTC conditions. However, contrary to our expectation, the population proportion of conformists were lowest under the moderate RTC condition rather than the high RTC condition. Yet, as expected, it is under the high RTC condition that conformists were most likely to be pushed into the marginalized social spaces of society and become more like the deviants, especially when diversity among conformists is high. These patterns for conformists make sense in light of the idea that high RTC conditions are maintained at the cost of reducing the available social space in the mainstream of society, thereby pushing a greater number of conformists into the marginalized social space of society compared to if RTC was low. Furthermore, as diversity within the conformists increases, more conformists will either be influenced by the deviants in the marginalized social space they are pushed into or even be more like the deviants to begin with. Thus, the population proportion of conformists is maximized when resistance to change is low and when diversity is low. This suggests that diversity can be a detriment to maintaining a healthy population of conformists who can enjoy the access to the necessary institutional means for accomplishing cultural goals, such as having a stable income and being able to easily raise a family, etc. Thus, it appears that diversity among conformists may mean that conformists are more likely to either be treated as another class of citizens or cross over the boundaries into another class, especially into the marginalized class of deviants.

### 5.2. Rebels

We anticipated the patterns for rebels to be similar to those of deviants, at least as a function of RTC and Diversity levels. Our expectations were not confirmed. In fact, it was almost exactly the opposite. That is, the population proportions of rebels were smallest, rather than the largest, in the high RTC condition relative to the other RTC conditions, which were themselves no different from each other. Also, the population proportion of rebels was largest, rather than smallest, in the high diversity condition relative to the other diversity conditions, where no differences were seen. Similarly, the interaction effect between RTC and diversity on the population proportion of rebels was also in the exact opposite direction than expected. That is, the population proportion of rebels was smallest, rather than the largest, in the combined condition of high RTC and low diversity. These patterns for rebels suggest that rebels generally thrive under RTC and diversity conditions in which the population proportions of deviants are minimized rather than maximized. Consequently, contrary to our expectations, it is unlikely that conditions that create deviants are the same conditions that create rebels. If anything, rebels are more likely to emerge under conditions of institutional equilibrium rather than institutional disequilibrium. Put another way, the current ABM suggests that there is more room to rebel against institutions and dominant cultural goals the more diversity there is in society and the less society's institutions are resistant to change. Therefore, we were wrong in speculating that the emergence of rebels is generally a response to increasing likelihood of being marginalized in society. Our model, instead, suggests that this is generally not the case. Marginalization does not always drive rebellion.

These results appear at first glance to be counterintuitive. But they are borne out by some of the political science literature on the emergence of rebellions. Benjaminsen (2008), for example, says that the Tuareg rebellion in Northern Mali was preceded by “a strong feeling among nomads and Tuareg in Mali of being marginalized by state policies of modernization.” He concludes that droughts played a role in the rebellion but were not the driving force. Rather, the droughts forced young Tuareg men to flee to Algeria and Libya, where they were exposed to “revolutionary discourse.” That is to say, they were exposed to cultural ideas that were not in the air in their homeland. A plausible retelling of the story says that the Tuareg rebels became rebels when they were able to consider ideas different from the ones present in their own cultural spaces. That is, greater cultural diversity was the kindling that sparked the men to transform from alienated farmers to rebels.

Having said that, rebellion is not more likely to occur under conditions where conformists thrive either. While both conformists and rebels thrive under lower RTC conditions, rebels thrive under higher diversity conditions even though high diversity can be detrimental to maintaining a healthy population proportion of conformists. Thus, rebellion may be a response to perceived intolerance for deviant behaviors and norm violations, rather than a response to inequality of opportunities.

### 5.3. Quiet mavericks

Quiet mavericks represent a blend of conformists and rebels. They are the more innovative and creative individuals in society who can flexibly conform to the expectations and norms of their society while also being able to effect change from inside the system to create more opportunities for others. These are the silicon valley-type entrepreneurs, social entrepreneurs, and organizational leaders who know how to effectively put new and innovative ideas into action. While it seems obvious that these individuals would have to have had access to institutional means to accomplish cultural goals, as the conformists do, what makes them different from conformists may be their unique experiences and unique characteristics. Thus, it was not clear to us if the conditions of RTC and diversity would systematically relate to the population proportion of quiet mavericks. Consequently, our examination of the patterns of quiet mavericks as a function of RTC and diversity levels was not hypothesis-driven but simply exploratory.

The results of the ABM suggest that RTC and Diversity do play important roles with respect to quiet mavericks. Specifically, the population proportion of quiet mavericks was larger in the low RTC condition relative to the other RTC conditions, which were no different from each other. Also, the population proportion of quiet mavericks was largest in the high diversity condition and lowest in the low diversity condition with the moderate diversity condition falling in between. There was no interaction between RTC and diversity on population proportion. It should make sense that within group diversity would increase the likelihood for quiet mavericks to emerge. However, what was particularly interesting is that conformists were more likely to become quiet mavericks under the low RTC condition and, separately (i.e., no interaction effect), under the high diversity condition. This suggests that there is a link between conformists and quiet mavericks, at least under certain conditions. Specifically, the more conformists interacted with quiet mavericks under low RTC conditions and under high diversity condition, the more likely some of those conformists will become transformed into quiet mavericks. However, when RTC is high or when diversity is low, quiet mavericks are more likely to remain an isolated group of individuals from the other classes of individuals and not grow too much in size.

### 5.4. Methodological issues

As with all ABMs, this work is limited by the fact that the data are obtained from a computer simulation that examines how micro-level interactions accumulate and translate into macro-level changes over time. While the ABM is the only known way to directly manipulate micro-level processes to examine macro level changes over time, it is by definition, not an empirical study but a theoretical one. So one may question the generalizability of ABM findings to the real world. Nevertheless, it is a specific application of our empirically grounded theory of the psychology and sociology of marginalization (see Norasakkunkit and Uchida, 2011, 2014; Norasakkunkit et al., 2012) to examine how the theory plays out under specified parameters defined by RTC and Diversity levels. It is also based on educated guesses (from sociological knowledge) with respect to the starting point for the population proportions of different classes of agents and the likelihood that they would interact with each other under varying RTC and Diversity levels. So ABMs can be epistemically useful even if they don't capture all possible variables^12^.

While the ABM is also limited by our capacity to imagine all the relevant independent variables that could go into the model, we were intentionally interested in examining the role that RTC and Diversity play on the cultural changes of different classes of individuals in society, given that our previous theoretical work (Toivonen et al., 2011; Norasakkunkit and Uchida, 2012; Norasakkunkit et al., 2012) had already suggested that RTC and diversity would play crucial roles in impacting the degree to which a large segment of society may be constrained from participating in the mainstream of society and therefore become marginalized. Thus, at least the hypotheses that were relevant to conformists, deviants, and how they relate to each other under varying RTC and diversity conditions, were firmly grounded in previous empirical and theoretical work. In contrast, we had little empirical and theoretical grounding to generate specific hypotheses with respect to rebels. Therefore, perhaps the patterns of findings with respect to rebels should have not been hypothesized but simply explored, just as the patterns of findings with respect to quiet mavericks were examined for exploratory purposes.

Another methodological issue concerns modeling membership in a culture. We chose to allow agents to remain a member of their own cultures until they possessed the 8 traits needed to become a member of another culture. However there are several other ways to capture membership in a culture as well as becoming a member of a new culture. We'll discuss one we believe is particularly interesting. Let's call an agent who is partly a member of a culture a “partial member.” One way to accommodate partial members is to create an entirely new category of agents. We'll call them “Misfits” since they don't quite fit anywhere. Misfits can consist of all of those agents who have fewer than some critical number of traits needed to become a member of a culture. A Misfit, for example, might have three Rebel traits or two Conformist traits or both. This agent doesn't pass for either a Rebel or a Conformist. She is excluded by both. Throwing into one pool all the agents who do not belong elsewhere creates a hodgepodge of traits. It is possible that a Misfit culture emerges; equally possible is that no set of traits emerges. Future ABMs will likely bear out an answer.

We opted not to pursue this option in our model because Misfits do not appear in Merton's taxonomy. Nonetheless, the existence of Misfits is a live possibility, the implications of which can be explored through ABMs.

A final concern about our model concerns hones in on how we have modeled marginalization and subcultures. A population is marginalized to the extent that is has insufficient access to social resources. We've represented that in terms of share of the population of deviants. Deviants are, by definition, the ones with the least access to resources for achieving goals. We've represented their inability to access those resources in terms of degree of overlap with Conformists. Deviants initially don't share any traits in common with Conformists. Over 2.5 generations, they come to share some traits, and they are the most similar to one another under the conditions of High RTC and Low Diversity and least similar under conditions of Low RTC and High Diversity. However, another plausible way in which to capture marginalization might be to focus on wealth-gaps between the über-wealthy and the rest of the population. Access to resources is determined by, among other things, economic standing. The more money one has, the greater the access to resources like quality education and health care. So in some post-industrial societies, a minority of the population might have the greatest access to the resources. In American culture, this is manifested in the correlation between wealth and political influence: wealthier individuals are able to have disproportionate influence on elections. Gilens and Page's (2014) study of American policy decisions between 1981 and 2002 show that policies tend to follow the desires of the economic elites^13^. The key question to our mind is: how ought we to represent access to resources? Surely, an American family earning $125,000/year has access to resources not available to a family earning $30,000/year. But neither of these families has the kind of access bought by an income of $10 million. In a sense, the family earning $125,000 a year is marginalized because they lack the kind of political influence owned by the über-wealthy: the rich-but-not-wealthy family is fortunate that the über-wealthy's preferences work in their favor. There are two ways in which our model is able to accommodate this phenomenon with minor tweaks. First, the über-wealthy are members of the population of conformists and the RTC value needs only to be tightened. This would readily accommodate the phenomenon here into the model. What would not be represented is the disproportionate influence by a minority of Conformists. That it, Conformists as a whole would be influential in maintaining social norms. But among Conformists, a small number would be doing the lion's share of work. Second, the über-wealthy might be modeled as their own culture with the RTC set very low. Again, this would capture the disproportionate influence that this subgroup would have on the population as a whole. The über-wealthy's recalcitrance would eventually bring the traits of other cultures into line with their own.

## 6. Conclusion

Our model tells a story linking resistance to change and cultural tightness to marginalization. When the culture is highly resistant to change and culturally tight, then those who have the least in common with the mainstream are the most likely to be marginalized. And when the opposite holds—when the culture is open to change and diverse, then fewer people end up in marginalized spaces. We did not expect Deviants to be most similar to Conformists under conditions of low Diversity. The Deviant and Conformist subcultures began with nothing in common. And low Diversity conditions makes overlap on secondary traits less likely than high Diversity.

One subculture in the model about which little is known is Quiet Mavericks. Our model suggests that they flourish when RTC is low and Diversity is high. Future research with the model might provide insight into the cultural influences of Quiet Mavericks as they begin to take up more of the population.

## Author contributions

CL: created agent-based model, wrote sections describing model; VN: primary writer of sections on background theory, conducted data analysis; BS: debugged agent-based model, proofreader; TT: contributed to sections on background theory.

### Conflict of interest statement

The authors declare that the research was conducted in the absence of any commercial or financial relationships that could be construed as a potential conflict of interest.

## Footnotes

^1^In recent years, philosophers of science have wondered whether such simplified models are useful for understanding such complex phenomena (cf. Weisberg, 2012). For our part, we believe that the simplified model enables users to witness interactions of two important macro-level properties: diversity and resistance to change. Clearly, our model can't be the final word on the matter, for we've only picked out two of many macro-level properties. However, we believe our model offers insights into the macro conditions that lead to marginalization of groups over time. For understanding how diversity and resistance to change lead to marginalization lays the groundwork for understanding how other local socio-cultural properties interact with those macros to create marginalized groups.

^2^In a Durkheimian sense, “anomie” refers to the breakdown of social bonds between an individual and his/her cultural community.

^3^We chose 2.5 generations as our target timeframe because notable cultural shifts can happen in as little time as that in post-industrial societies.

^4^Short for *O*verview, *D*esign concepts, *D*etails.

^5^For a selection of papers that have employed the ODD protocol, see citations in Grimm et al. (2010).

^6^“Breed” is not here used with any biological implications. Thanks to an anonymous reviewer for bringing this to our attention.

^7^One constraint on the model is that the population size is fixed at 200 agents rather than letting the overall population change over time. We chose to simplify this because we wanted to examine shifts in population proportions of the model that were the results of changes in RTC and Diversity. Analysis of this would have to control for fluxes in subculture populations. So rather than build that flux into the model only to control for it, we kept the populations size of the model fixed. Thanks to an anonymous reviewer for pressing for clarification on this point.

^8^These values are from Toivonen (personal communication).

^9^Thanks to an anonymous reviewer for suggesting we add this.

^10^At the start, the maximum value for *n* = 199, but as agents die off and more are born, the value of n increases.

^11^Five features with ten traits had on average 3.2 stable regions. Ten and fifteen features with fifteen traits per feature had on average 1.4 and 1.2 stable regions, respectively.

^12^O'Connor, C. *Modeling Minimal Condition for Inequity*. Available online at: http://philsci-archive.pitt.edu/13473

^13^Gilens and Page define “economic elite” as someone in the top 90th percentile, which is $146,000 and above. They note that someone at this level of income isn't “wealthy” in the same way that someone in the 98th percentile is wealthy. But they show that political preferences of the über-wealthy track those at the 90th percentile. So while there is no existing data on the political preferences of the über-wealthy, the data tracking preferences of the top 10% is a suitable proxy. In fact, they note that this method likely underestimates the influence of the über-wealthy.
